# Effect of the Seasonal Climatic Variations on the Accumulation of Fruit Volatiles in Four Grape Varieties Under the Double Cropping System

**DOI:** 10.3389/fpls.2021.809558

**Published:** 2022-01-27

**Authors:** Hao-Cheng Lu, Wei-Kai Chen, Yu Wang, Xian-Jin Bai, Guo Cheng, Chang-Qing Duan, Jun Wang, Fei He

**Affiliations:** ^1^Center for Viticulture and Enology, College of Food Science and Nutritional Engineering, China Agricultural University, Beijing, China; ^2^Key Laboratory of Viticulture and Enology, Ministry of Agriculture and Rural Affairs, Beijing, China; ^3^Guangxi Academy of Agricultural Sciences, Nanning, China; ^4^Grape and Wine Research Institute, Guangxi Academy of Agricultural Sciences, Nanning, China

**Keywords:** climate factors, double cropping system, table grape, volatile compounds, wine grape

## Abstract

The double cropping system has been widely applied in many subtropical viticultural regions. In the 2-year study of 2014–2015, four grape varieties were selected to analyze their fruit volatile compounds in four consecutive seasons in the Guangxi region of South China, which had a typical subtropical humid monsoon climate. Results showed that berries of winter seasons had higher concentrations of terpenes, norisoprenoids, and C6/C9 compounds in “Riesling,” “Victoria,” and “Muscat Hamburg” grapes in both of the two vintages. However, in the “Cabernet Sauvignon” grapes, only the berries of the 2014 winter season had higher terpene concentrations, but lower norisoprenoid concentrations than those of the corresponding summer season. The Pearson correlation analysis showed the high temperature was the main climate factor that affected volatile compounds between the summer and winter seasons. Hexanal, γ-terpinene, terpinen-4-ol, *cis*-furan linalool oxide, and *trans*-pyran linalool oxide were all negatively correlated with the high-temperature hours in all of the four varieties. Transcriptome analysis showed that the upregulated *VviDXSs*, *VviPSYs*, and *VviCCDs* expressions might contribute to the accumulations of terpenes or norisoprenoids in the winter berries of these varieties. Our results provided insights into how climate parameters affected grape volatiles under the double cropping system, which might improve the understanding of the grape berries in response to the climate changes accompanied by extreme weather conditions in the future.

## Introduction

The grape double cropping system has been applied widely in many subtropical regions (Favero et al., 2011; Chou and Li, 2014). The traditional single cropping system seems not applicable in these regions because of the excess heat resources and the heavy rainfall in the summer season. The excessive rainfall and temperature during the grape ripening period can easily cause insufficient fruit ripeness and fungal infections. Moreover, relatively high temperature in winter does not meet the low-temperature requirements of the grapes for their normal dormancy, which results in uneven bud bursts in the spring (Favero et al., 2011). However, if the dormant buds were forced out of dormancy early during the current season, the double cropping system could be achieved (Chen et al., 2017). Even in winter, there was still adequate temperature and sunlight for the berry ripening in the subtropical viticulture regions, making the double cropping system more commercially adopted. There were two advantages of applying the double cropping system in these regions: (1) in the summer season, the grape berries could ripen earlier than the normal single cropping system, which could avoid the intense rainfall and heatwave as much as possible; and (2) in the winter season, cool climate and less rainfall usually led to better grape quality (Xu et al., 2011; Chen et al., 2017).

In the double cropping system, bud break was usually enforced between late January and mid-February in the northern hemisphere, resulting in the first bloom in early April and the first crop in June or July. Vines were then pruned and forced again around mid-August, resulting in the second bloom of the side shoots in mid-September and the second crop in mid-January of the following year (Chou and Li, 2014). Even in the one-crop-a-year culture system, berries composition could vary significantly in different vintages (Downey et al., 2006). In the double cropping system, the climate variations between the summer season and winter season were greater than the single cropping system, which led to great variations in grape qualities. The winter berries were usually considered more favorable for wine production than the summer berries. Junior et al. (2017) showed that the higher values of yield, cluster weight, and titratable acidity (TA) were observed during the summer growing season, whereas the higher values of soluble solids content and pH were observed during winter, which suggested that the grapes harvested during the winter show physicochemical characteristics more suitable than those observed during the summer crops for winemaking purposes in Brazil. However, the winter berries usually had lower cluster weights than the summer berries, thus leading to a lower yield in the winter season (Mitra et al., 2018). Some previous researches reported that the fruitfulness of the second crop of some cultivars, such as “Summer Black,” was much worse in some subtropical areas (Guo et al., 2018). Some plant growth inhibitors, such as chlormequat chloride (CCC), were usually used to promote inflorescence induction to enhance fruitfulness.

For grape secondary metabolites, the phenolic compositions were the focus of many researchers in dissecting the variations between the summer and the winter berries in the double cropping system (Xu et al., 2011; Chen et al., 2017; Zhu et al., 2017; Cheng et al., 2019). Similar results were found by previous studies that phenolic compounds, including anthocyanins, flavonols, and flavan-3-ols, were significantly higher in the winter season berries than in the summer season berries. Chen et al. (2017) showed that winter season berries greatly triggered the expression of the upstream genes in the flavonoid pathway in a coordinated expression pattern. However, other secondary metabolites were little studied in the double-cropping system, such as volatile compounds.

Volatile compounds are critical secondary metabolites in grapes, which play an essential role in their sensory evaluations. Aromas derived from grapes mainly include norisoprenoids, terpenes, C6/C9 compounds, methoxypyrazines, etc. (Wang et al., 2020). Terpenes and norisoprenoids have low sensory thresholds and pleasant flavors (Fenoll et al., 2009). Grapes of the Muscat family usually have abundant terpenes, which contribute to their intense varietal flavors. Commonly identified terpenes in grapes include a rose oxide, geraniol, nerol, linalool, terpineol, and citronellol, which contribute to the typical rose and floral aroma (Fenoll et al., 2009). 1,1,6-Trimethyl-1,2-dihydronaphthalene (TDN), β-damascenone, and β-ionone are common C_13_-norisoprenoids that contribute to fruity, violet, and petrol aromas to grapes and wines (Black et al., 2015). The abundant C6/C9 compounds in grapes contribute to a typical “green leaf” aroma, so they are also called green leaf volatiles (GLVs) (Kalua and Boss, 2010). The metabolic pathways of these volatile compounds are complicated, and many of them are still not quite clear until now. Mevalonic acid (MVA) and 2-methyl-D-erythritol-4-phosphate phosphate (MEP) pathway, carotenoid metabolism, and oxylipin pathway were the most investigated pathways, which could synthesize terpenes, norisoprenoids, and C6/C9 compounds, respectively (Kalua and Boss, 2009; Miziorko, 2010; Lashbrooke et al., 2013). The grape aromas were not only affected by the varieties and their development periods but also affected by the climate factors, such as temperature and light. In general, cluster exposure was beneficial for the accumulation of terpenes, whereas shading would reduce terpene concentrations (Bureau et al., 2000; Zhang et al., 2017). Grapes in cool-climate regions usually had higher C6 aldehyde concentrations, whereas warm-region grapes usually had higher terpene concentrations (Wen et al., 2015; Xu et al., 2015b). Rainfall and irrigation also affected the aroma accumulation in grapes. Regulated deficit irrigation during the berry development would promote the accumulation of terpenes (Savoi et al., 2016).

In a previous study, we investigated the variations of ripening progression and flavonoid metabolism in Cabernet Sauvignon (CS) and Riesling (R) grapes under the double cropping system (Chen et al., 2017). In the present study, the aroma characteristics in grapes under the double cropping system were furthermore investigated. Moreover, the two wine grape varieties, another two table grape varieties, Muscat Hamburg (MH) and Victoria (V), that occupied a good market in South China were also investigated. There were significant climate variations between the summer and winter seasons, and the corresponding aroma variations were also found in all of the four varieties under the double cropping system. This study helped us to understand better how climate parameters affected grape volatiles under the double cropping system, which might improve the understanding of the grape berries in response to the climate changes accompanied by extreme weather conditions in the future. Furthermore, the feasibility of applying a double-cropping system in viticulture in South China could be evaluated.

## Materials and Methods

### Experiment Site and Double Cropping System

The 2-year (2014–2015) study was performed at Guangxi Academy of Agricultural Sciences located in South China (22°36′N–108°14′E, elevation 104 m). The climate belonged to a subtropical humid monsoon climate with abundant sunshine and heat resources. Vines were trained to a Y-shaped training system with 2 × 4/5 shoots per meter and 1.0 m cordon above ground. Rain shelters were applied to all vines to prevent over-rainfall damage. Four varieties were investigated in this study: CS, R, MH, and V. CS and R grapevines were in the same vineyard, whereas MH and V grapes were in another. The distance between the two vineyards was within 1 km. The detailed information on the variety and phenological stages are shown in Supplementary Tables 1, 2. Clusters were weighted at harvest, and estimated yield was obtained by multiplying the average cluster weight by the average cluster numbers per meter.

The double cropping system in the experiment site was described by Chen et al. (2017). Briefly, vines were pruned two times, and the grapes were harvested two times per year. In mid-February, 2.5–3.0% hydrogen cyanamide was used to accelerate the bud burst. Summer grapes were harvested around late July and early August, which was called the summer cropping cycle. Then vines were pruned and followed the same procedure in August to start the second season. Winter grapes were harvested in January, which was called the winter cropping cycle.

### Berry Sampling and Meteorological Data Collection

Berries of all varieties were sampled four times in each growing season. Sampling time points were as follows: (1) pea-size (E-L 31), (2) onset of veraison (E-L 35), (3) veraison complement (E-L 36), and (4) harvest (E-L 38). There were three biological replicates for each variety. For each replicate, 300 berries were randomly sampled from about 50 vines, which were distributed in three adjacent rows. One hundred berries of which were used in the determination of the physicochemical parameters. The remaining berries were immediately frozen in liquid nitrogen and stored at −80°C for the subsequent metabolite and transcriptome analysis.

Meteorological data was acquired from the local climate monitoring station within 1 km away from the experiment site. Photosynthetically active radiation and temperature were recorded per hour. Accumulated rainfall was recorded per day. Growing degree days (base 10°C) was calculated from bloom to harvest according to Bindi et al. (1997).

### Analysis of Grape Physicochemical Parameters

For each replicate, 100 berries were weighted and then manually squeezed. The must was centrifuged (8000 × *g*) to get clear juice. The total soluble solids (TSS) of the juice were determined with a digital pocket handheld refractometer (PAL-1, ATAGO CO., LTD., Tokyo, Japan). The juice pH was determined by a pH meter (Sartorius PB-10, Gottingen, Germany). TA was measured and expressed as tartaric acid equivalents (g/L) according to the National Standard of People’s Republic of China (GB/T15038-2006).

### Extraction of Grapes Volatile Compounds

The extraction of grapes volatile compounds was according to Lan et al. (2016). For each replicate, about 50 g of berries were de-seeded and mashed under liquid nitrogen. Then, the frozen samples with the addition of 1 g polyvinylpolypyrrolidone and 0.5 g D-gluconic acid lactone were ground into powder. The frozen powder was melted under 4°C for about 6 h and then centrifuged at 8000 × *g* to get the clear juice. For free volatile compounds, 5 ml grape juice was added in a 20-ml vial with 1 g NaCl and 10 μl 4-methyl-2-pentanol (internal standard). For bound volatile compounds, 2 ml of the clear grape juice sample was added to Cleanert^®^ PEP-SPE resins (150 mg/6 mL, Bonna-Agela Technologies, Tianjin, China), which had been activated with 10 ml of methanol and 10 ml of water. Then, the resins were washed with 2 ml of water and 5 ml of dichloromethane to remove water-soluble compounds and free volatiles, respectively. The resins were eluted with 20 ml methanol afterward. The methanol extract was concentrated to dryness by a rotary evaporator under vacuum at 30°C and was redissolved in 10 ml of citrate/phosphate buffer solution (0.2 M, pH = 5). The enzymatic hydrolysis of glycosidic precursors was conducted at 40°C for 16 h by adding 100 μl AR 2000 (Rapidase, 100 g/L, DSM Food Specialties, France). The 5 ml of the above sample was added in a 20-ml vial with 1 g NaCl and 10 μl 4-methyl-2-pentanol (internal standard). Both free and bound samples were placed in a CTC-Combi PAL autosampler (CTC Analytics, Zwingen, Switzerland) equipped with a 2-cm DVB/CAR/PDMS 50/30 μm SPME fiber (Supelco Inc., Bellefonte, PA., United States) and agitated at 500 rpm for 30 min at 40°C. The SPME fiber was then inserted into the headspace to absorb aroma compounds at 40°C for 30 min and was instantly desorbed into the gas chromatography (GC) injector for 8 min to thermally desorb aroma compounds, and the injection temperature was set at 250°C.

### Gas Chromatography–Mass Spectrometer Analysis of Volatile Compounds in Grapes

Both free-volatile and bound-form aroma compounds were extracted by headspace solid-phase microextraction (HS-SPME). Agilent 6890 GC coupled with Agilent 5973C mass spectrometer (MS) was used for the aroma determination. GC was equipped with an HP-INNOWAX capillary column (60 m × 0.25 mm, 0.25 μm, J&W Scientific, Folsom, CA, United States) to separate volatile compounds. The carrier gas was high purity helium with a flow rate of 1 ml/min. The oven program was set as follows: 50°C for 1 min, increased to 220°C at a rate of 3°C/min, and held at 220°C for 5 min. Identification and quantification of volatile compounds followed our research group method (Wang et al., 2019). Concentrations of volatile compounds were expressed as μg/L grape juice.

### RNA Extraction and Transcriptome Sequencing

The berries of three development stages (E-L 35, 36, and 38) in 2014 were selected to determine the transcriptome sequencing. For each replicate, de-seeded 50 berries were ground into powder under liquid nitrogen protection. The following procedures had been described by Chen et al. (2017). Briefly, the sample total RNA was extracted by using a Spectrum Plant Total RNA Kit (Sigma-Aldrich, Carlsbad, CA, United States). Transcriptome analysis was conducted on the Illumina HiSeq 2000 platform (Illumina, Inc., San Diego, CA, United States) with 50-bp single reads and aligned against the reference grapevine genome 12 × V2, allowing no more than two mismatches. Gene expression abundance was calculated using the fragments per kilobase per million reads (FPKM) method to eliminate the influence of variation in gene length and total reads numbers on gene expression calculation (Sun et al., 2015). The R package “DESeq2” was used to identify differentially expressed genes (DEGs), and the criteria were set as false discovery rate ≤ 0.05 and fold change ≥ 2. Gene Ontology (GO) and Kyoto Encyclopedia of Genes and Genomes (KEGG) enrichment analysis of DEGs was used to select candidate genes responsible for the differences in aroma compounds between seasons. The data have been deposited in the NCBI Gene Expression Omnibus (GEO) database and are accessible through GEO accession GSE103226 (CS and R grapes) and GSE168785 (V and MH grapes). Total reads and total mapped reads per sample are shown in Supplementary Table 3.

### Statistical Analysis

The SPSS version 22.0 (SPSS Inc., United States) was used for all significance analysis at *p* < 0.05 (Duncan’s multiple range test or *t*-test). The Pearson correlation analysis was performed in MataboAnalyst 4.0^1^. The figures were prepared by using GraphPad Prism 8.0.2 (GraphPad Software, San Diego, CA, United States), SIMCA 14.1 (Umetrics, Umea, Sweden), and R-3.6.1. Heatmap was prepared by using the “pheatmap” package in R. Principal component analysis (PCA) was performed in SIMCA 14.1 (Umetrics, Umea, Sweden).

## Results

### Meteorological Data

Meteorological data in the year 2014 and 2015 were shown and discussed by Chen et al. (2017). The climate conditions of growing seasons in all varieties were further analyzed in this study (Table 1). CS and R grapes had a similar phenological stage in all growing seasons in 2014 and 2015. The MH and V grapes had a similar phenological stage in the winter seasons of 2014 and 2015. In the summer season of 2015, MH grapes were harvested 23 days later than V grapes. The summer season had higher mean daily temperature and more high-temperature hours than the winter season in all varieties, but the cumulative PAR and rainfall were not consistent in the years 2014 and 2015. For CS and R grapes, the winter season had less cumulative PAR but similar cumulative sunshine hours and rainfall than the summer season in 2014. In 2015, the summer season of CS and R grapes had more cumulative PAR, sunshine hours, and rainfall than the winter season. In 2014, the winter season of MH and V grapes had more cumulative PAR, sunshine hours, and rainfall than the summer season, while in 2015, the winter season of MH and V grapes had fewer cumulative PAR and sunshine hours. Furthermore, the summer season of MH had more rainfall than the winter season. The weather condition in 2010–2019 was analyzed to present the regular climate characteristics in Guangxi (Supplementary Figure 1). June, July, and August were the hottest months in the whole year with almost the most abundant rainfall, which was a typical subtropical humid monsoon climate. However, the rainfall and sunshine hours also had wide ranges in many months, which made a high intra- or interseasonal variability.

**TABLE 1:** Meteorological data in 2014 and 2015 growing seasons.

Year	Season	Variety	GDD (°C)	Average daily temperature (°C)	High temperature (>35°C) hours	Cumulative PAR (10^3^ μmol/m^2^/s)	Cumulative sunshine hours (h)	Cumulative rainfall (mm)
2014	Summer	CS and R	2128.6	28.8	459	45.9	483.3	488.2
	Summer	MH and V	1588.9	28.7	351	33.9	312.2	226.5
	Winter	CS and R	1042.9	19.7	109	41.0	503.2	466.7
	Winter	MH and V	1123.2	19.3	89	38.4	506.3	503.5
2015	Summer	CS and R	2180.8	29.3	490	54.1	586.5	599.8
	Summer	V	1699.6	29.7	410	45.2	475.7	386.0
	Summer	MH	2238.8	29.7	516	56.5	627.1	599.8
	Winter	CS and R	1330.4	20.6	62	40.4	384.0	398.3
	Winter	MH and V	1207.0	20.3	45	39.2	379.1	378.4

*GDD, growing degree days (based on 10°C); CS, Cabernet Sauvignon; R, Riesling; MH, Muscat Hamburg; V, Victoria; PAR, photosynthetically active radiation.*

### Grape Physicochemical Parameters

The physicochemical parameters of CS and R grapes were shown and discussed by Chen et al. (2017). In brief, the berries in the summer cropping showed a higher TSS at E-L 31 but showed an opposite result at E-L 38 in CS and R grapes. Berry weight increased along with the berry development, but the berry weight in the winter cropping was significantly lower than those of the summer cropping at E-L 35, 36, and 38 in CS and R grapes. The TSS, TA, pH, and 100 berries weight of MH and V grapes in different sampling times are shown in Table 2. In 2014, the winter season berries had higher TSS than the summer season berries during the development stages. V grapes reached only 12.8°Brix at harvest in the summer season of 2014, which was almost 8°Brix lower than their corresponding winter season berries. In 2015, there was no significant difference in TSS of MH berries between the summer and winter seasons during the development stages. For V grapes, the summer season grapes ripened faster than the winter season ones, although there was still higher TSS in winter season berries at harvest. MH and V berries had lower TA and higher pH in the summer season than the winter season. Similar to CS and R grapes, berry weight in the winter cropping was also lower than that in the summer season in MH and V grapes. Reduced berry weight in the winter cropping led to a lower yield than the summer cropping (Supplementary Table 2).

**TABLE 2:** Physicochemical parameters of MH and V grapes grown under double cropping system in 2014 and 2015.

Parameters	Development stages	MH		Sig.	V		Sig.	MH		Sig.	V		Sig.
		2014 Summer	2014 Winter		2014 Summer	2014 Winter		2015 Summer	2015 Winter		2015 Summer	2015 Winter	
TSS (Brix)	E-L 31	3.7 ± 0.1	4.4 ± 0.6	ns	4.0 ± 0.2	3.7 ± 0.5	ns	5.5 ± 0.9	5.4 ± 0.6	ns	5.7 ± 0.3	4.6 ± 0.3	*
	E-L 35	8.3 ± 2.0	12.3 ± 0.2	*	9.1 ± 0.3	12.8 ± 1.3	*	8.0 ± 1.9	7.4 ± 0.2	ns	9.1 ± 0.3	6.5 ± 0.2	*
	E-L 36	17.4 ± 0.8	19.0 ± 0.9	ns	12.6 ± 0.4	18.3 ± 0.5	*	17.6 ± 1.1	16.4 ± 0.4	ns	15.4 ± 0.6	11.6 ± 0.6	*
	E-L 38	19.8 ± 1.3	22.0 ± 0.4	*	12.8 ± 0.8	20.7 ± 0.5	*	20.4 ± 0.8	20.9 ± 1.0	ns	15.6 ± 0.5	17.0 ± 0.5	*
TA (g/L)	E-L 31	27.3 ± 0.5	30.0 ± 3.3	ns	35.1 ± 1.1	28.3 ± 5.3	ns	33.3 ± 5.8	31.4 ± 1.3	ns	30.0 ± 1.5	24.4 ± 2.3	*
	E-L 35	11.8 ± 1.0	20.6 ± 1.7	*	6.8 ± 1.2	10.2 ± 1.2	*	27.6 ± 1.8	33.9 ± 0.2	*	6.8 ± 1.2	26.7 ± 1.8	*
	E-L 36	5.8 ± 0.3	12.0 ± 2.0	*	2.3 ± 0.0	6.7 ± 0.7	*	2.2 ± 0.1	10.4 ± 1.1	*	2.0 ± 0.4	6.1 ± 0.8	*
	E-L 38	3.7 ± 0.4	8.1 ± 0.3	*	1.8 ± 0.1	3.9 ± 0.2	*	1.8 ± 0.3	5.1 ± 0.2	*	1.4 ± 0.2	2.5 ± 0.3	*
pH	E-L 31	2.48 ± 0.04	2.41 ± 0.08	ns	2.41 ± 0.06	2.35 ± 0.05	ns	2.33 ± 0.01	2.40 ± 0.02	*	2.32 ± 0.03	2.26 ± 0.06	ns
	E-L 35	2.64 ± 0.25	2.60 ± 0.03	ns	3.14 ± 0.11	2.86 ± 0.05	*	2.50 ± 0.01	2.41 ± 0.01	*	3.14 ± 0.11	2.37 ± 0.03	*
	E-L 36	3.47 ± 0.03	3.18 ± 0.11	*	4.02 ± 0.09	3.35 ± 0.10	*	4.15 ± 0.03	2.99 ± 0.07	*	4.17 ± 0.08	3.17 ± 0.06	*
	E-L 38	3.85 ± 0.17	3.42 ± 0.08	*	4.12 ± 0.10	3.89 ± 0.09	*	4.34 ± 0.09	3.46 ± 0.12	*	4.36 ± 0.06	3.83 ± 0.08	*
100 berries weight (g)	E-L 31	124.9 ± 17.0	107.0 ± 8.9	ns	209.1 ± 9.9	153.1 ± 14.5	*	172.1 ± 2.4	169.9 ± 11.6	ns	180.4 ± 4.7	74.4 ± 5.0	*
	E-L 35	267.8 ± 17.9	155.0 ± 7.5	*	536.4 ± 7.3	309.2 ± 19.6	*	236.7 ± 16.6	205.4 ± 7.3	*	362.6 ± 45.5	251.4 ± 24.4	*
	E-L 36	320.2 ± 4.3	159.6 ± 10.8	*	723.4 ± 20.3	371.8 ± 27.0	*	378.2 ± 35.1	315.7 ± 9.3	*	792.8 ± 10.8	582.5 ± 30.9	*
	E-L 38	361.4 ± 8.8	265.8 ± 16.9	*	734.2 ± 137.3	408.6 ± 31.0	*	372.6 ± 14.3	357.3 ± 19.1	ns	786.1 ± 29.6	578.4 ± 60.7	*

*MH, Muscat Hamburg; V, Victoria; TSS, total soluble solids; TA, titratable acidity; 2014 Summer, 2014 summer season; 2014 Winter, 2014 winter season; 2015 Summer, 2015 summer season; 2015 Winter, 2015 winter season. Values are reported as mean ± SD of three biological replicates. Sig., significance. Asterisk indicates there are significant differences between summer and winter season berries (p < 0.05, t-test). ns, not significant.*

### Grape Volatile Compounds

**FIGURE 1:**
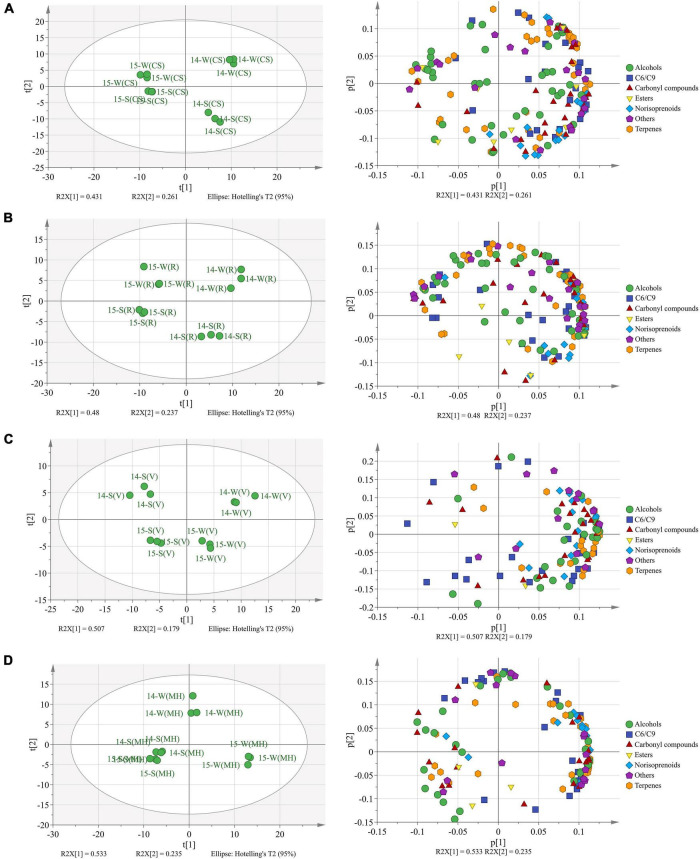
The principal component analysis (PCA) based on the aroma compounds in Cabernet Sauvignon **(A)**, Riesling **(B)**, Victoria **(C)**, and Muscat Hamburg **(D)** grapes in 2014 and 2015. 14-S, 2014 summer season; 14-W, 2014 winter season; 15-S, 2015 summer season; 15-W, 2015 winter season.

Totally, there were 173 free-form and 137 bound-form volatile compounds identified in the four grape varieties, and these compounds were sorted into seven groups: C6/C9 compounds, terpenes, norisoprenoids, alcohols, carbonyl compounds, esters, and others (Supplementary Tables 4, 5). The PCA was used to identify the aroma profile variations between the mature grapes (E-L 38) under the double cropping system, as shown in Figure 1.

In CS grapes (Figure 1A), two principal components explained 69.2% of the total variance. PC1 (R2X[1]) discriminated the berries of 2014 from those of 2015 and accounted for 43.1% of the total variance. The loading plot showed that the CS berries of 2014 had more abundant aroma compounds than those of 2015. PC2 (R2X[2]) discriminated the winter berries from the summer berries and accounted for 26.1% of the total variance. The loading plot showed that the winter berries had abundant terpenes, and the summer berries had abundant norisoprenoids.

In R grapes (Figure 1B), two principal components explained 71.7% of the total variance. Similar to the CS grapes, the two principal components could discriminate berries of the four seasons from each other. The loading plot showed that the winter berries had more abundant aroma compounds than the summer berries, especially terpenes.

In V grapes (Figure 1C), two principal components explained 68.6% of the total variation. PC1 (R2X[1]) separated the berries of different seasons, accounted for 50.7% of the total variation. Vintage variation only occupied 17.9% of the total variation. The winter season berries had an abundant aroma profile than the summer season berries, and most terpenes and norisoprenoid concentrations were higher in the winter berries than those in the summer berries.

In MH grapes (Figure 1D), two principal components explained 76.8% of the total variation. Unlike the previous three varieties, the summer season berries of 2014 and 2015 could not be clearly discriminated by the PCA model. PC1 (R2X[1]) accounted for 53.3% of the total variation that could discriminate the winter season berries of 2015 from the berries of the two summer seasons. PC2 (R2X[2]) accounted for 23.5% of the total variation that could discriminate the winter season berries of 2014 from other seasons. The winter season berries of 2015 had the most abundant aroma compounds, especially terpenes, norisoprenoids, and C6/C9 compounds.

#### Total Concentrations of C6/C9 Compounds, Terpenes, and Norisoprenoids

**FIGURE 2:**
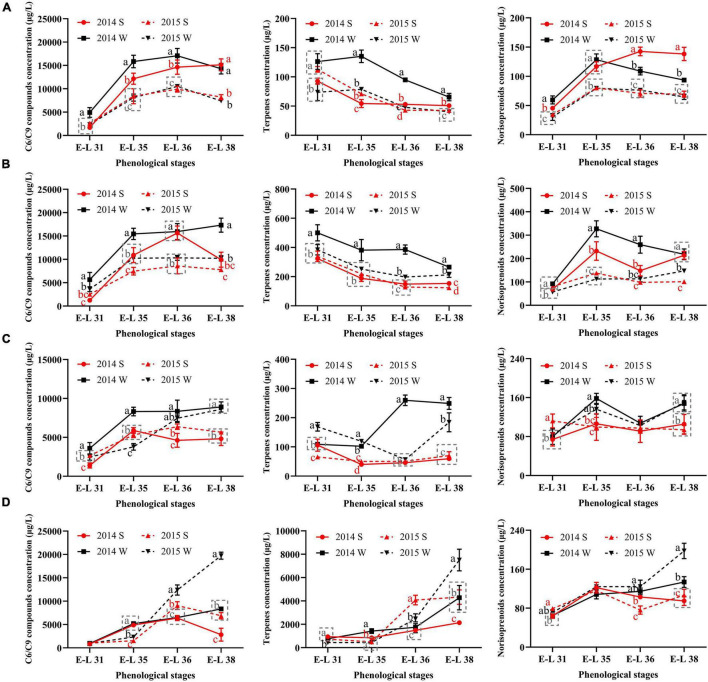
The total concentrations of C6/C9 compounds, terpenes, and norisoprenoids in Cabernet Sauvignon **(A)**, Riesling **(B)**, Victoria **(C)**, and Muscat Hamburg **(D)** grapes in 2014 and 2015. 2014 S, 2014 summer season; 2014 W, 2014 winter season; 2015 S, 2015 summer season; 2015 W, 2015 winter season. Different letters indicate significant differences among seasons based on Duncan’s multiple range test at *p* < 0.05.

To figure out how the grape-derived aromas changed during the development stages, the total concentrations of C6/C9 compounds, terpenes, and norisoprenoids were calculated, as shown in Figure 2.

The C6/C9 compounds were the most abundant aroma compounds in all the grapes. The accumulation trends of C6/C9 compounds were not consistent in the four growing seasons. In most seasons, C6/C9 compounds peaked at E-L 36 and then declined until the harvest, which was in agreement with the previous study (Wang et al., 2019). However, there were some seasons when the grapes of E-L 38 had the highest C6/C9 concentration, such as the winter season of V and MH grapes. In CS grapes, the berries of 2014 had higher C6/C9 compound concentrations than those of 2015. The significant difference between the summer and winter seasons in C6/C9 compound concentrations only occurred in the former development stages in 2014. However, in the other three varieties, the winter season berries had higher C6/C9 compound concentrations than those of the summer season within the same vintage in most development stages, especially at harvest.

For terpenes, MH grapes had the highest concentration among the four varieties, with at least 2000 μg/L at harvest. The other three varieties only had 50–400 μg/L terpenes at harvest, indicating that the grapes of the Muscat family usually had abundant terpenes (Fenoll et al., 2009). CS and R grapes had similar trends in terpenes accumulation. They had the highest total terpene concentrations at E-L 31, then declined until harvest. In MH grapes, the highest terpene concentration occurred at E-L 38. In V grapes, a significant increase of terpene only occurred in the 2014 winter season as grapes developed. However, in other seasons, the terpene concentration at harvest was slightly higher or have little difference than E-L 31 in V grapes.

For norisoprenoids, there were no consistent results in all varieties. In CS grapes, the summer season berries of 2014 had higher norisoprenoid concentration than those of the winter season in the same year, whereas no significant difference showed in the vintage of 2015. In R grapes, the significant differences between the summer and winter season berries occurred at E-L 35 and E-L 36 in 2014 and at E-L 38 in 2015. MH and V grapes had the same results in the two vintages, and the winter berries had higher norisoprenoid concentrations than those of the summer season.

### Variations of Volatile Compounds Between Growing Seasons

**FIGURE 3:**
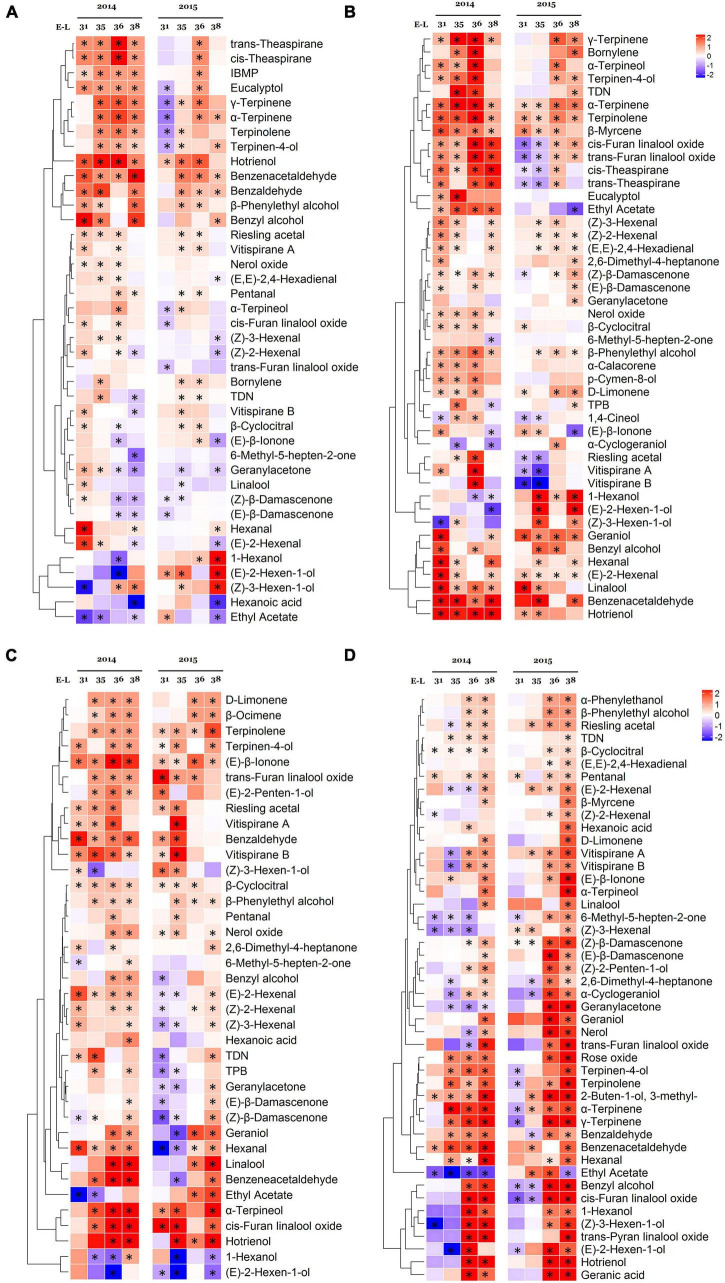
Effect of the growing season on the free volatile compounds in Cabernet Sauvignon **(A)**, Riesling **(B)**, Victoria **(C)**, and Muscat Hamburg **(D)** grapes during fruit development in 2014 and 2015. Heatmaps show the log_2_ fold changes between seasons (winter season/summer season). Red block indicates higher aroma concentrations in the winter season berries. Blue block indicates lower aroma concentrations in the winter season berries. *Significant differences between the summer and winter season (*p* < 0.05, *t*-test). IBMP, 2-isobutyl-3-methoxypyrazine; TDN, 1,1,6-trimethyl-1,2-dihydronaphthalene; TPB, (*E*)-1-(2,3,6-trimethylphenyl)buta-1,3-diene.

To figure out how the growing seasons affected the concentrations of individual volatile compounds, the key compounds were selected by using the *t*-test, which showed significant differences in at least one sampling point between seasons. The selected free volatile compounds are shown in Figure 3.

In CS grapes, many terpenes had higher concentrations in the winter season in several or a specific stage, especially in 2014. However, some norisoprenoids, such as (*E*)-β-ionone, 6-methyl-5-hepten-2-one, geranylacetone, (*Z*)-β-damascenone, and (*E*)-β-damascenone had lower concentrations in the winter season. β-Damascenone occupied the highest proportion in norisoprenoids of CS grapes (Supplementary Table 4). (*E*)-2-hexenal and hexanal were the main C6/C9 compounds with the highest concentrations (Supplementary Table 4). The winter berries had higher hexanal concentration but had lower (*E*)-2-hexenal than the summer berries at the harvest date.

In R grapes, most terpenes had higher concentrations in the winter season, such as γ-terpinene, α-terpinene, β-myrcene, terpinolene, geraniol, etc. In 2014, many terpenes only had higher concentrations before harvest in the winter season berries, but these differences disappeared at harvests, such as bornylene, α-terpineol, terpinen-4-ol, α-calacorene, and D-limonene. Although most norisoprenoids had higher concentrations in several stages, (*E*)-β-ionone was the only norisoprenoid with a higher concentration in the summer berries in both of the two vintages at harvest. TDN was well-known to contribute “petrol” aromas to “Riesling” wines (Sacks et al., 2012), and it had a higher concentration in the winter berries at harvest in 2015. Different from CS grapes, its winter season berries had high concentrations of both (*E*)-2-hexenal and hexanal.

In V grapes, most terpenes were also more abundant in the winter season berries, such as D-limonene, β-ocimene, terpinolene, terpinen-4-ol, linalool, α-terpineol, *cis*-furan linalool oxide, etc. For norisoprenoids, the winter season berries had a higher (*E*)-β-ionone concentration in all sampling times of 2014 and 2015. (*Z*)-β-damascenone and (*E*)-β-damascenone only showed higher concentrations in the winter season berries at harvest in 2014 and 2015. Similar to R grape, its (*E*)-2-hexenal and hexanal also had higher concentrations in the winter season berries at harvest.

In MH grapes, almost all of the selected compounds had higher concentrations in the winter season berries at E-L 38. Many of them did not show any difference in the early development, even were higher in the summer season berries at E-L 35 and E-L 36.

Except for terpenes, norisoprenoids, and C6/C9 compounds, some benzene derivatives were also had higher concentrations in the winter season berries in all varieties, such as benzaldehyde, benzeneacetaldehyde, benzyl alcohol, and β-phenylethyl alcohol. These compounds contribute roasted, honey, almond, fruity, and floral flavors to the grapes or their wines (Cai et al., 2014). High temperatures could enhance their biotransformation and degradation rate, whereas lower temperatures would increase their concentrations (Scafidi et al., 2013).

The selected bound volatile compounds are shown in Supplementary Figure 2. Compared to the free volatile compounds, there were fewer selected bound-form compounds with significant differences between seasons. In CS grapes, most of the selected compounds had no consistent trends in 2014 and 2015. Only 2,3-butanedione, *cis*-furan linalool oxide, and *trans*-furan linalool oxide showed higher concentrations in the winter season berries in serval stages over 2 years. In R and V grapes, most of the terpenes had higher concentrations in the winter season berries, which was in agreement with the free volatile results. However, in MH grapes, most of the selected compounds showed the opposite trends in 2014 and 2015.

### Relationship Between Volatile Compounds and Climate Factors

As mentioned above, the summer seasons had more high-temperature hours than the winter seasons, but the accumulated PAR and rainfall were not consistent in the two vintages. Thus, the Pearson correlation analysis was used to figure out how climate factors affect the berries’ volatile compounds at harvest. The highly correlated compounds (| *r*^2^| > 0.6) in at least three varieties were selected, as shown in Table 3. Seventeen volatiles showed high correlations to the high-temperature hours, and most of them were negatively correlated. For C6/C9 compounds, hexanal was negatively correlated with the high temperatures in all of the four varieties. For terpenes, γ-terpinene, terpinen-4-ol, *cis*-furan linalool oxide (G), and *trans*-pyran linalool oxide (G) were all negatively correlated with high temperatures in all of the four varieties. Other terpenes, such as geraniol, were negatively correlated with high temperatures in R, MH, and V grapes, and geranial (G), nerol (G), geraniol (G), and *p*-cymene were negatively correlated with high temperatures in R and MH grapes. Different from the pronounced effects of high-temperature hours on aromas, fewer compounds highly correlated with PAR and rainfall were selected. Free- and bound-form *p*-menthan-8-ol concentrations were all positively correlated with accumulated whole season PAR. *trans*-Furan linalool oxide (G) was negatively correlated with accumulated whole season PAR in CS, R, and MH grapes. The rainfall correlated compounds had no consistent trends in four varieties.

**TABLE 3:** The Pearson correlation analysis between grape volatile compounds and climate factors.

Climate factor	Compound	Correlation			
		CS	R	MH	V
High-temperature hours	(*E,E*)-2,4-hexadienal	0.20	–0.65	−0.87*	–0.60
	(*E*)-2-hexen-1-ol	–0.62	0.15	0.68**	–0.69
	Hexanal	–0.43	–0.70	−0.91*	−0.87*
	γ-Terpinene	–0.46	–0.61	−0.91*	−0.96*
	Terpinen-4-ol	–0.67	–0.49	−0.95*	−0.87*
	Geraniol	0.45*	−0.75*	−0.84*	−0.79*
	1-Octen-3-ol	–0.30	−0.78*	–0.68	−0.79*
	Benzeneacetaldehyde	–0.58	–0.70	−0.78*	−0.92*
	*cis*-Furan linalool oxide (G)	–0.74	−0.96*	−0.84*	–0.73
	*trans*-Pyran linalool oxide (G)	−0.77*	−0.99*	−0.76*	−0.80*
	α-Ionene	0.64**	0.10	–0.73	−0.92*
	Geranylacetone	0.71**	0.05	−0.82*	–0.67
	Geranial (G)	−	−0.79*	−0.78*	0.81**
	Nerol (G)	0.21	−0.76*	−0.79*	0.82**
	Geraniol (G)	0.08	−0.90*	−0.81*	0.73**
	1-Heptanol (G)	0.12	−0.82*	−0.83*	0.62**
	*p*-Cymene (G)	−	−0.85*	−0.85*	0.73**
Accumulated whole-season PAR	*p*-Menthan-8-ol	0.65**	0.89**	−	0.86**
	*trans*-Furan linalool oxide (G)	−0.75*	−0.78*	–0.61	0.40
	*p*-Menthan-8-ol (G)	0.92**	0.92**	−	0.86**
	3-Buten-1-ol, 3-methyl-(G)	0.29	−0.83*	0.64**	0.86**
Accumulated whole-season rainfall	1-Octen-3-ol	−0.75*	−0.82*	0.45*	0.76**
	α-Terpineol (G)	–0.56	−0.78*	0.63**	0.63**
	*trans*-Pyran linalool oxide (G)	–0.70	−0.77*	0.12	0.64**
	Acetoin (G)	−0.87*	–0.07	−0.78*	0.72**

CS, Cabernet Sauvignon; R, Riesling; MH, Muscat Hamburg; V, Victoria. (G), glycosidically bound form. –, not detected. **Significantly correlated at p < 0.01; *significantly correlated at p < 0.05.

### Expression Profiles of Aroma Synthesis-Related Genes in the Grapes

#### 2-Methyl-D-Erythritol-4-Phosphate Phosphate and Mevalonic Acid Pathway

**FIGURE 4:**
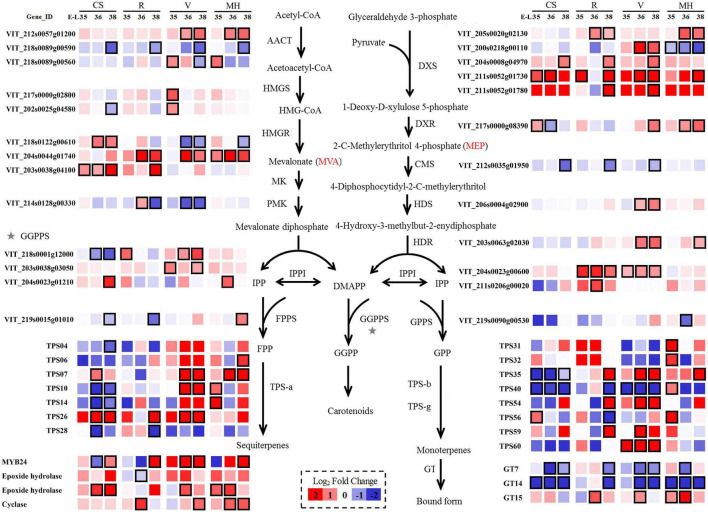
Effect of the growing season on the expression profiles of MEP and MVA synthesis pathways during Cabernet Sauvignon, Riesling, Victoria, and Muscat Hamburg grapes development in 2014. Heatmaps show the log_2_ fold change between the seasons (winter season/summer season). Red block indicates higher gene expression in the winter season berries. Blue block indicates lower gene expression in the winter season berries. Boxes with bold margins indicate differently expressed genes between the summer and winter season berries. MEP, 2-methyl-D-erythritol-4-phosphate phosphate; MVA, mevalonic acid.

Terpenes were derived from two common precursors: isopentenyl pyrophosphate (IPP) and its isomer dimethylallyl diphosphate (DMAPP), which were synthesized from two independent pathways: the plastidial MEP and the cytoplasmic MVA pathways, respectively (Wen et al., 2015). Carotenoids, the precursors of norisoprenoids, were also synthesized from the MEP pathway (Meng et al., 2020). The log_2_ fold change was used to present the variations between the summer and winter season berries through MVA and MEP pathways (Figure 4).

In the MEP pathway, five *VviDXS* genes (VIT_200s0218g00110, VIT_204s0008g04970, VIT_205s0020 g02130, VIT_211s0052g01730, and VIT_211s0052g01780) were expressed differently in the berries of the winter and summer seasons in at least two varieties. Only the expression of *VviDXS2* (VIT_200s0218g00110) was downregulated in MH grapes, and other genes were all upregulated in several varieties in the winter season berries. *VviDXS3* (VIT_204s0008g04970) was the common upregulated expression gene in the winter season berries of all four varieties. *VviIPI* (VIT_204s0023g00600 and VIT_211s0206g00020) was responsible for the transformation between IPP and DMAPP, which had upregulated expression in R and V winter season berries. The mRNA levels of the GPPS small subunit might play a key role in regulating the formation of GPPS and thus affecting the monoterpene biosynthesis (Tholl et al., 2004). In the present study, *VviGPPS small subunit* (VIT_219s0090g00530) had higher expressions in MH grapes than other varieties and had low expression levels in CS and V grapes (Supplementary Table 6), which might be correlated with the monoterpene concentration variation among these varieties.

In the MVA pathway, there were also many genes significantly affected by the growing seasons. The expression of one *VviAACT* gene (VIT_218s0089g00590) was downregulated in the winter season berries of all four varieties. HMGR was the key enzyme of the MVA pathway. There were three *VviHMGR* genes (VIT_203s0038g04100, VIT_204s0044g01740, and VIT_218s0122g00610) differently expressed in the winter and summer season berries. Among them, VIT_204s0044g01740 was upregulated in R, V, and MH grapes. Terpene synthases (TPSs) were the final enzymes of the terpene biosynthetic pathway. *TPS-a*, *TPS-b*, and *TPS-g* were the main *VviTPS* genes with high expressions (Wen et al., 2015). In CS grapes, most selected *VviTPS* genes were downregulated in the winter season berries. Glycosyltransferases (GTs) could converse free terpenes into their corresponding glycoside bound forms, and three genes had been proved to have such character: *VviGT7*, *VviGT14*, and *VviGT15* (Bönisch et al., 2014). The downregulated expression of *VviGT7* and *VviGT14* were shown in all of the four varieties in the winter season berries.

#### Carotenoid Metabolism Pathway

As mentioned above, the MEP pathway also synthesized carotenoids, which were the precursors of norisoprenoids. The following pathway after synthesizing geranylgeranyl diphosphate (GGPP) was shown in Supplementary Figure 3 and Supplementary Table 7. The condensation of two GGPPs by phytoene synthase (PSY) formed phytoene, the first carotenoid (Cazzonelli and Pogson, 2010). There were three identified *VviPSYs* genes (VIT_212s0028g00960, VIT_206s0004g00820, and VIT_204s0079g00680), which expressed differently between the berries of the summer and the winter season. *VviPSY3* (VIT_206s0004g00820) and *VviPSY2* (VIT_212s0028g00960) had higher expressions in the winter season berries and showed significant differences in several stages. Carotenoid cleavage dioxygenases (CCDs) were the key enzymes that catalyzed the generation of norisoprenoids (apocarotenoids) by cleaving the conjugate double bond of carotenoids (Meng et al., 2020). There were four *VviCCDs* selected in the present study, and most of them had higher expressions in the winter season berries, especially *VviCCD4a*. In CS grapes, *VviCCD4a* (VIT_202s0087g00910) had higher expressions at E-L 35 and E-L 38, whereas it was downregulated at the E-L 36 stage.

#### Oxylipin Pathway

The C6/C9 compounds, or GLVs, were short-chain alcohols, aldehydes, and esters formed through the oxylipin pathway (Hassan et al., 2015). The main enzymes in the oxylipin pathway included lipoxygenase (LOX), hydroperoxide lyase (HPL), and alcohol dehydrogenase (ADH). The selected genes were expressed differently in the oxylipin pathway between the summer and winter season berries are shown in Supplementary Figure 4. Compared with LOXs, *VviLOXA* (VIT_206s0004g01510) had high expression levels in the whole development stages of all the four varieties, which might play a key role in the LOX family (Supplementary Table 8; Podolyan et al., 2010; Xu et al., 2015a). The expression of *VviLOXA* was upregulated in the winter season berries of R, V, and MH grapes, whereas it was downregulated in the CS winter season berries. *VviHPL1* (VIT_212s0059g01060) had high expression levels in the development stages. It was reported that *VviHPL1* was also related to the accumulation of C6 compounds (Xu et al., 2015a). However, there were no consistent trends in all of the four varieties in the present study. ADH was responsible for the conversion of aldehydes to alcohols. About half of *VviADH* expressions were downregulated in the winter season berries in all of the four varieties, and others were upregulated. For C6 alcohols, only the MH winter season berries had higher (*E*)-2-hexen-1-ol and (*Z*)-3-hexen-1-ol concentrations than the summer season berries in 2014 and 2015. The winter season berries of CS and R had higher (*E*)-2-hexen-1-ol concentrations than the summer season berries in 2015 and showed an opposite trend in 2014. The V winter season berries had a lower (*E*)-2-hexen-1-ol concentration than the summer season berries in both of the two vintages.

#### Relationship Between Volatile Compounds and Transcriptome Gene Expression

To figure out the relationship between volatile compounds and transcriptome gene expression, we selected the transcriptome genes involved in C6/C9, terpenes, and norisoprenoids synthesis pathway to calculate their correlation with the concentration of each corresponding category during berry development (E-L 35, E-L 36, and E-L 38). The highly correlated compounds (the Pearson correlation analysis, | *r*^2^| > 0.6) in at least two varieties were selected, as shown in Supplementary Table 9. Only three genes involved in the oxylipin pathway showed high correlations to total C6/C9 compound concentration, and two of them were negatively correlated. The genes related to terpenes occupied the highest proportion in all selected genes, and 51 genes showed high correlations with total terpene concentration. Among them, five genes (VIT_211s0052g01730, VIT_203s0038g03050, VIT_202s0025g04864, VIT_202s0025g04880, and VIT_205 s0051g00670) were positively correlated with the total terpene concentration and six genes (VIT_215s0046g03550, VIT_215s0046g03590, VIT_215s0046g03600, VIT_215s0046 g03650, VIT_206s0004g02740, and VIT_214s0083g00770) were negatively correlated with high total terpene concentration in at least three varieties. The *VviDXS3* (VIT_211s0052g01730), which was upregulated in the winter berries in all varieties (Figure 4), was positively correlated with the berry terpene concentration in CS, V, and MH grapes. In the carotenoid metabolism pathway, only five genes were selected to have a high correlation with berry norisoprenoid concentration. Among them, two *VviCCDs* (VIT_213s0064g00810 and VIT_213s0064g00840) were positively correlated with the norisoprenoid concentration in R and V grapes, which was in agreement with the previous analysis. However, the two *VviCCDs* (VIT_213s0064g00810 and VIT_213s0064g00840) were upregulated in the winter berries in R and V grapes.

## Discussion

### Effect of the Growing Season on Berries Physicochemical Parameter

The berries in the summer season usually had lower TSS than in the winter season under the double cropping system (Xu et al., 2011; Zhu et al., 2017), which was also confirmed in the present study. Severer high-temperature pressure and fewer sunshine hours in the 2014 summer season might inhibit TSS accumulation in the grapes. For MH and V grapes, there were 351 high-temperature hours in the 2014 summer season but only 89 h in the 2014 winter season. Although elevated temperature usually accelerated the sugar accumulation in the grape berries, scorching conditions would exceed the optimum photosynthetic temperature (Gutiérrez-Gamboa and Moreno, 2019). When the temperature exceeded 35°C, it would cause damage to the photosynthetic apparatus of the grapevines (Gutiérrez-Gamboa et al., 2021). However, in 2015, there was no significant difference in TSS in the MH berries between the summer and winter seasons during the developmental stages. This might be due to the sunshine hours in the winter season of MH grape, which were only 57% of the summer season and led to less carbon assimilation of vines. Fewer sunshine hours during the grape development in the 2015 winter season might slow down the TSS accumulation in the V grapes, which led to a slower ripening rate from the stages of E-L 31 to E-L 35 in the winter season berries.

### Effect of the Growing Season on Berries Volatile Compounds

The winter season berries had higher terpene concentrations than those of the summer seasons in all of the four varieties. In general, most studies on the aromas and aroma precursors of fruity and floral nuances not only highlighted the benefit of the higher temperatures during berry ripening but also their negative effects on the fruit metabolism whenever they were excessively high (Pons et al., 2017). Grapes in warm regions were reported to have higher terpene concentrations than in hot regions (Lecourieux et al., 2017). The Pearson correlation analysis showed that γ-terpinene, terpinen-4-ol, *cis*-furan linalool oxide (G), and *trans*-pyran linalool oxide (G) were all negatively correlated with high temperatures in all of the four varieties in the present study. The elevated temperature (>35°C) would inhibit the accumulation of terpenes (Scafidi et al., 2013). Furthermore, terpene concentrations might be negatively correlated with the average daily maximum temperature during the ripening because of volatilization (Gutiérrez-Gamboa et al., 2021). In our study, the expression of *VviDXSs* was commonly upregulated in the winter season berries of all four varieties. Lecourieux et al. (2017) showed that the strong repression of the genes encoding the 1-deoxy-D-xylulose-5-phosphate synthase (VIT_05s0020g02130, VIT_09s0002g02050, VIT_11s0052g01730, and VIT_11s0052g01780) suggested that the volatile terpenoid biosynthesis might be decreased by high temperature. Similarly, Rienth et al. (2014) reported that high temperatures impaired the expression of 1-deoxy-D-xylulose-5-phosphate synthase transcripts (VIT_11s0052g01730 and VIT_11s0052g01780), which were required for the isopentenyl pyrophosphate (IPP) synthesis, the universal precursor for the biosynthesis of terpenes. The regression of high temperatures on the *VviDXSs* expression might be the reason for the lower terpene concentration in the summer season berries in the present study. There were also some gene expressions, such as *VviGTs*, that were downregulated in the winter season berries. The contents of many bound terpene substances are lower in winter than in the summer season (Supplementary Table 5 and Supplementary Figure 2). The GTs were responsible for the synthesis of bound terpene substances as a GT, so to a certain extent, it could be speculated that the downregulation of *VvGTs* expression caused a decrease of bound terpene substances in winter berries. Free- and bound-form *p*-menthan-8-ol concentrations were all positively correlated with the accumulation of PAR in the whole season. In general, previous studies reported that the increased light exposure was beneficial for the terpene accumulation, and the shading treatment led to lower monoterpenes levels in bunches (Bureau et al., 2000). In hot climates, the beneficial effect of increased synthesis of terpenes induced by light might be surpassed by the negative effect of the elevated berry temperature (Friedel et al., 2016). The rainfall correlated compounds had no consistent trends in all of the four varieties, and its effect might also be covered up by the high temperature effect.

The MH and V grapes had similar results in the two vintages, and the winter season berries had a higher norisoprenoid concentration than those of the summer season. Similar to the terpenes accumulation, high temperatures also inhibited the biosynthesis of norisoprenoids (Wang et al., 2020). Lecourieux et al. (2017) found that heat treatment would repress the expressions of the genes encoding the key enzymes in the carotenoid metabolism, which formed norisoprenoids. So in R, V, and MH grapes, the winter season berries had higher norisoprenoid concentrations than those of the summer seasons. Regarding the different results in CS grapes, different varieties had varied temperature, sunlight, or water requirements, leading to varying responses to the climate changes (Schultz, 2003; Parker et al., 2020). The CS grapes were reported as a late-ripening variety (Parker et al., 2013), which might require more temperature than the other three varieties. The winter seasons might not meet the temperature requirement for the norisoprenoids accumulation in CS grapes, which led to less norisoprenoid concentrations than in the summer seasons. Moreover, the variation between different vineyards might also contribute to the differences between CS and the other two table varieties. The east–west row orientation was believed to have the lowest sunlight interception in canopies among all vineyard orientations (Lu et al., 2021), which was also unfavorable for the norisoprenoid accumulation in CS grapes. In the present study, four *VviCCDs* were expressed differently in different growing seasons, and most of them had higher expressions in the winter season berries. Scherzinger and Al-Babili (2008) found that both cold (20°C) and heat stress (38°C) could increase the expression of the CCD genes. However, Meng et al. (2020) found the high temperature (37°C) repressed the activity of the *VvCCD4b* promoter. In the present study, the upregulated expression of most *VviCCDs* in the winter season berries might show that high temperatures were unfavorable for their expressions. However, in CS grapes, the winter season berries had lower norisoprenoid concentration than those in the summer season. Different expressions of *VviCCD1s* (VIT_213s0064g00840 and VIT_213s0064g00810) in CS grapes and other varieties might be the reason.

The accumulation trends of C6/C9 compounds were not consistent in the four growing seasons in the present study. In most seasons, C6/C9 compounds peaked at E-L 36 and then declined until the harvest. However, there were some seasons when the berries at E-L 38 had the highest C6/C9 concentrations, such as the winter seasons of V and MH grapes. Kalua and Boss (2010) found that CS grapes had the highest C6/C9 compound concentration at pre-harvest, whereas R grapes had the highest C6/C9 compound concentration at harvest. As the C6 compounds are derived from varietal precursors, they could hypothetically contribute to judging wine origin and affiliation (Oliveira et al., 2006). In the Pearson correlation analysis in this study, hexanal was negatively correlated with high temperatures in all of the four varieties. As reported, hexanal was derived from linoleic acid hydroperoxide through the LOX pathway (Oliveira et al., 2006). Podolyan et al. (2010) also reported that two recombinant LOXs had the maximum enzymatic activity at 25°C and lost about 40% of their maximal activity when the temperature exceeded 35°C. As for LOXs, the expression of *VviLOXA* was upregulated in the winter berries of R, V, and MH grapes, whereas it was downregulated in CS winter berries. R, V, and MH winter berries had higher C6/C9 concentrations than the summer berries, whereas there was no significant difference in CS grapes at harvest. The variations of *VviLOXA* expression in CS and other varieties might be the reason.

## Conclusion

This research used metabolomics and transcriptomics to reveal the aroma variations in different grape varieties under the double cropping system. The winter berries had higher TSS content and TA than the summer berries. The lower berry weight in the winter season caused a decreased yield compared to those of the summer season. The winter berries had higher concentrations in many aroma categories than the summer berries, especially terpenes. Climate factor variations were the main reason for the quality variations in the summer and winter season berries. Among all of the climate factors, the temperature might be the dominant one, and its influence could cover up the effects of other factors. Different from other varieties, the CS winter berries had lower norisoprenoid concentrations than the summer berries, indicating that the responses to climate changes might be variety-dependent. The higher concentrations of terpenes and norisoprenoids in the winter berries of most varieties could be associated with the regulated expressions of *VviDXSs*, *VviPSYs*, and *VviCCDs* at the transcription level. The different climates in the summer and winter seasons provided us with a better understanding of how climate changes influenced the grapes’ secondary metabolites. The season variation within a vintage under the double cropping system usually exceeded the effects of the vintage in the traditional viticulture regions, making our results more apparent.

## Data Availability Statement

The original contributions presented in the study are publicly available. This data can be found here: National Center for Biotechnology Information (NCBI) BioProject database under accession numbers GSE103226 and GSE168785.

## Author Contributions

FH, JW, and C-QD designed the experiments. W-KC and H-CL performed the experiments. H-CL analyzed the data and prepared the original draft. YW provided the statistical support. X-JB and GC provided the double cropping system vineyards. FH revised the manuscript. All authors approved the submitted version.

## Conflict of Interest

The authors declare that the research was conducted in the absence of any commercial or financial relationships that could be construed as a potential conflict of interest.

## Publisher’s Note

All claims expressed in this article are solely those of the authors and do not necessarily represent those of their affiliated organizations, or those of the publisher, the editors and the reviewers. Any product that may be evaluated in this article, or claim that may be made by its manufacturer, is not guaranteed or endorsed by the publisher.

## Footnotes

1
https://www.metaboanalyst.ca/
