# Supplementary material for: Effect of the Seasonal Climatic Variations on the Accumulation of Fruit Volatiles in Four Grape Varieties Under the Double Cropping System
Source: Front Plant Sci. 2022 Jan 27;12:809558. doi: 10.3389/fpls.2021.809558 (PMC8829325; doi:10.3389/fpls.2021.809558)
Supplement: Supplementary file 1 [file Data_Sheet_1.docx]

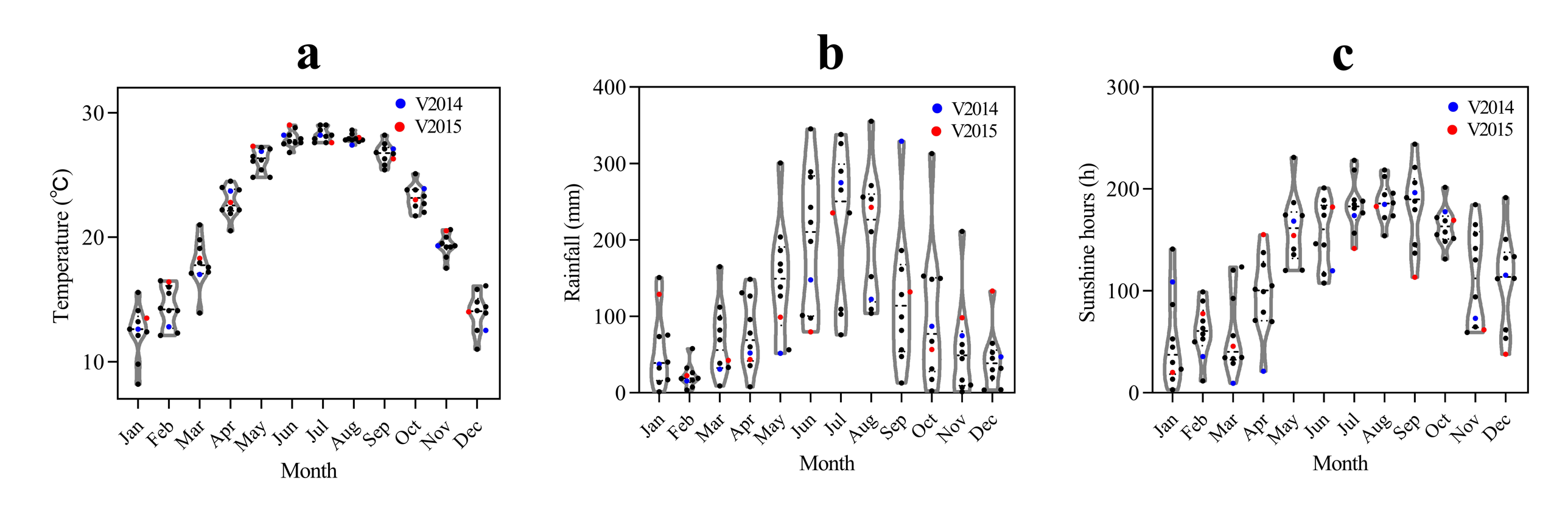


**Supplementary Figure 1.** Summary of monthly average meteorological data in the year of 2014 and 2015: (a) temperature (℃), (b) rainfall (mm), (c) sunshine hours (h). The combination of violin and point drawing revealed the distribution of the monthly data from 2010 to 2019. Blue point revealed the vintage 2014 (Feb. 2014- Jan. 2015). Red point revealed the vintage 2015 (Feb. 2015- Jan. 2016).


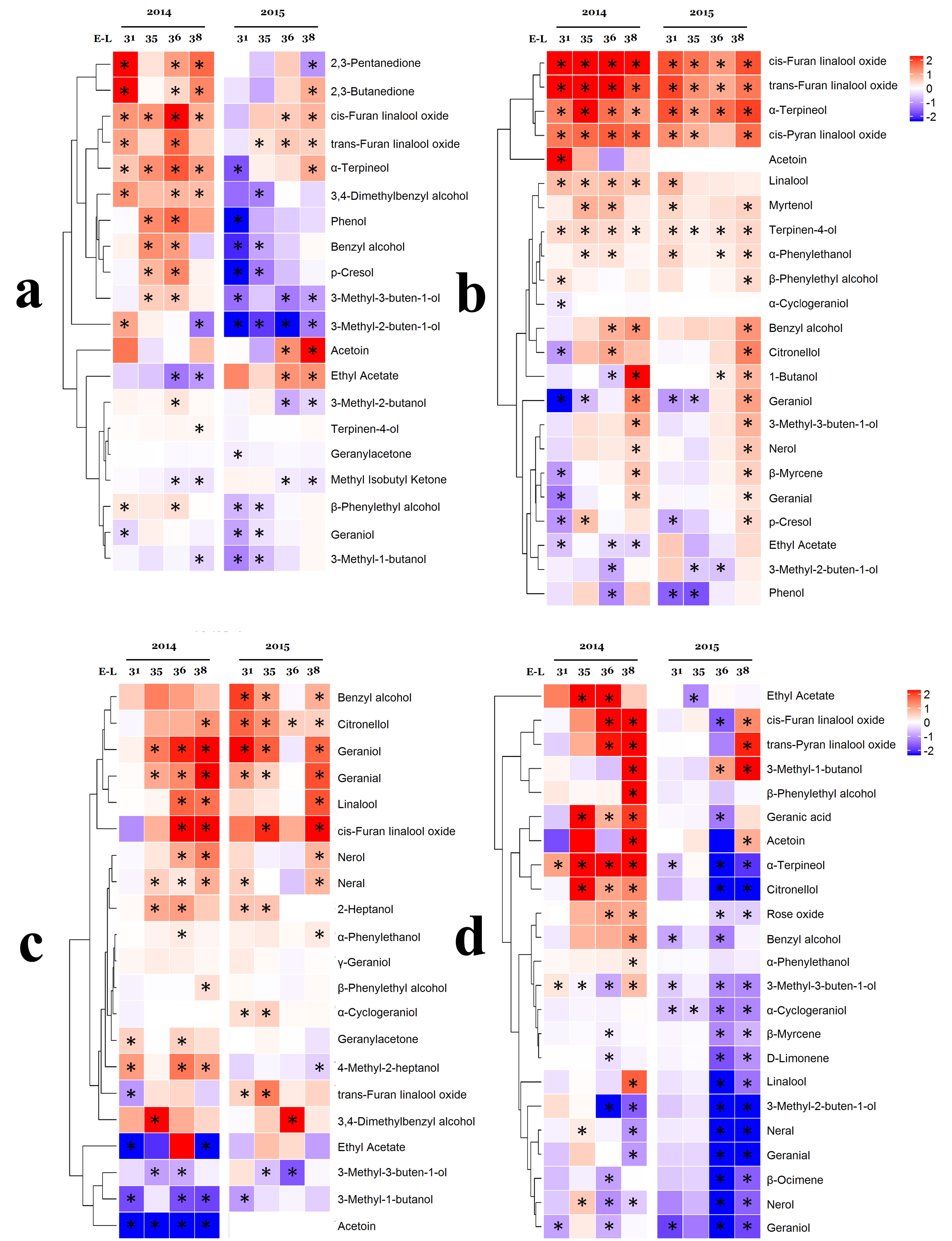


**Supplementary Figure 2.** Effect of the growing season on the bound form volatile compounds in CS (a), R (b), V (c), and MH (d) grapes during fruit development in the year of 2014 and 2015. Heatmaps showed the log_2_ fold change between seasons (winter season/summer season). Red block indicates higher aroma concentration in winter season berries. Blue block indicates lower aroma concentration in winter season berries. * indicates there are signiﬁcant differences between summer and winter season (*p* < 0.05, *t*-test).


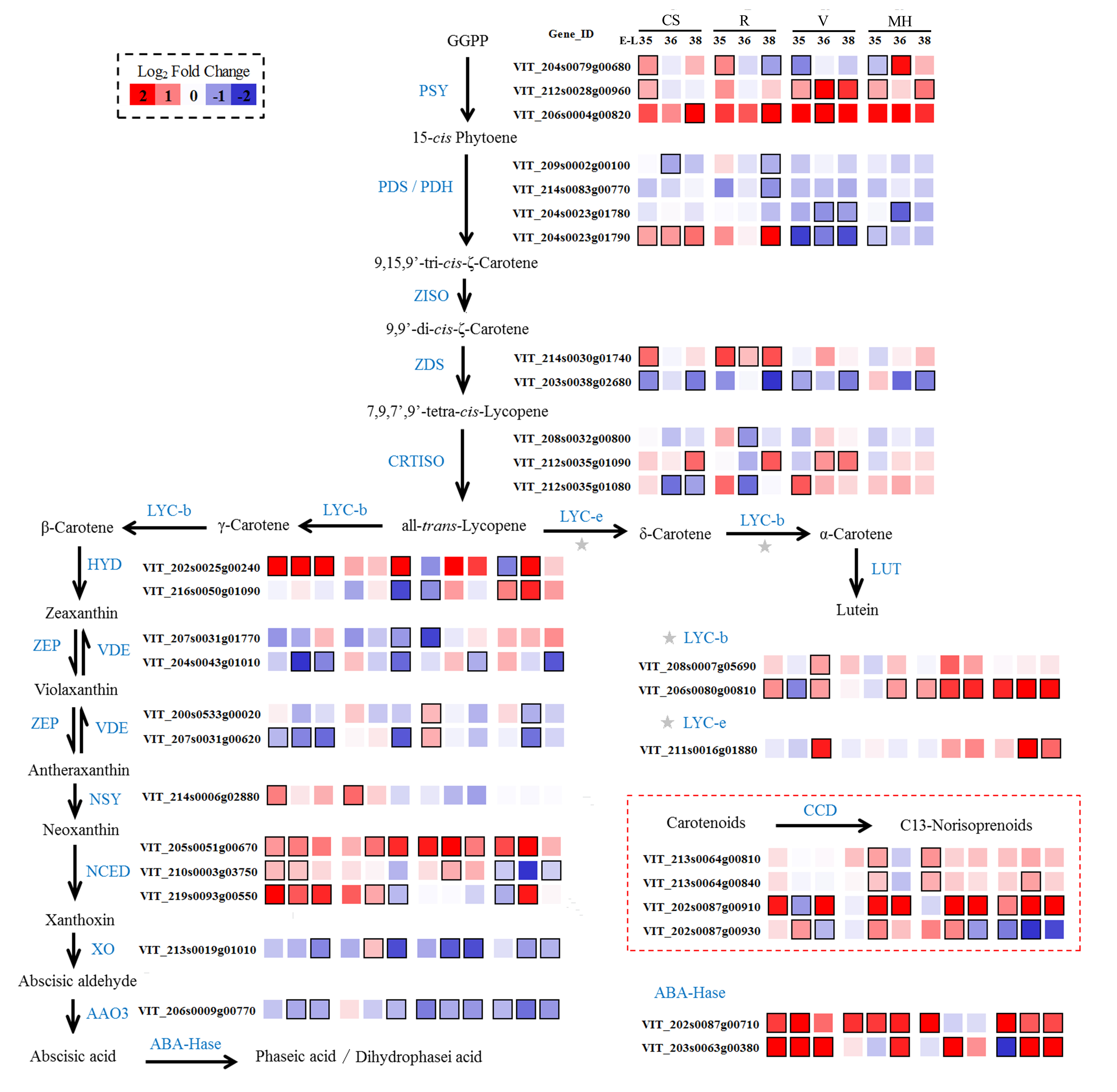


**Supplementary Figure 3.** Effect of the growing seasons on the expression profiles of carotenoid metabolism synthesis pathways during the development of CS, R, V, and MH grapes in 2014. Heatmaps showed the log_2_ fold change between seasons (winter season/summer season). Red block indicates higher gene expression in winter season berries. Blue block indicates lower gene expression in winter season berries. Boxes with bold margins indicate differentially expressed genes between summer and winter berries.


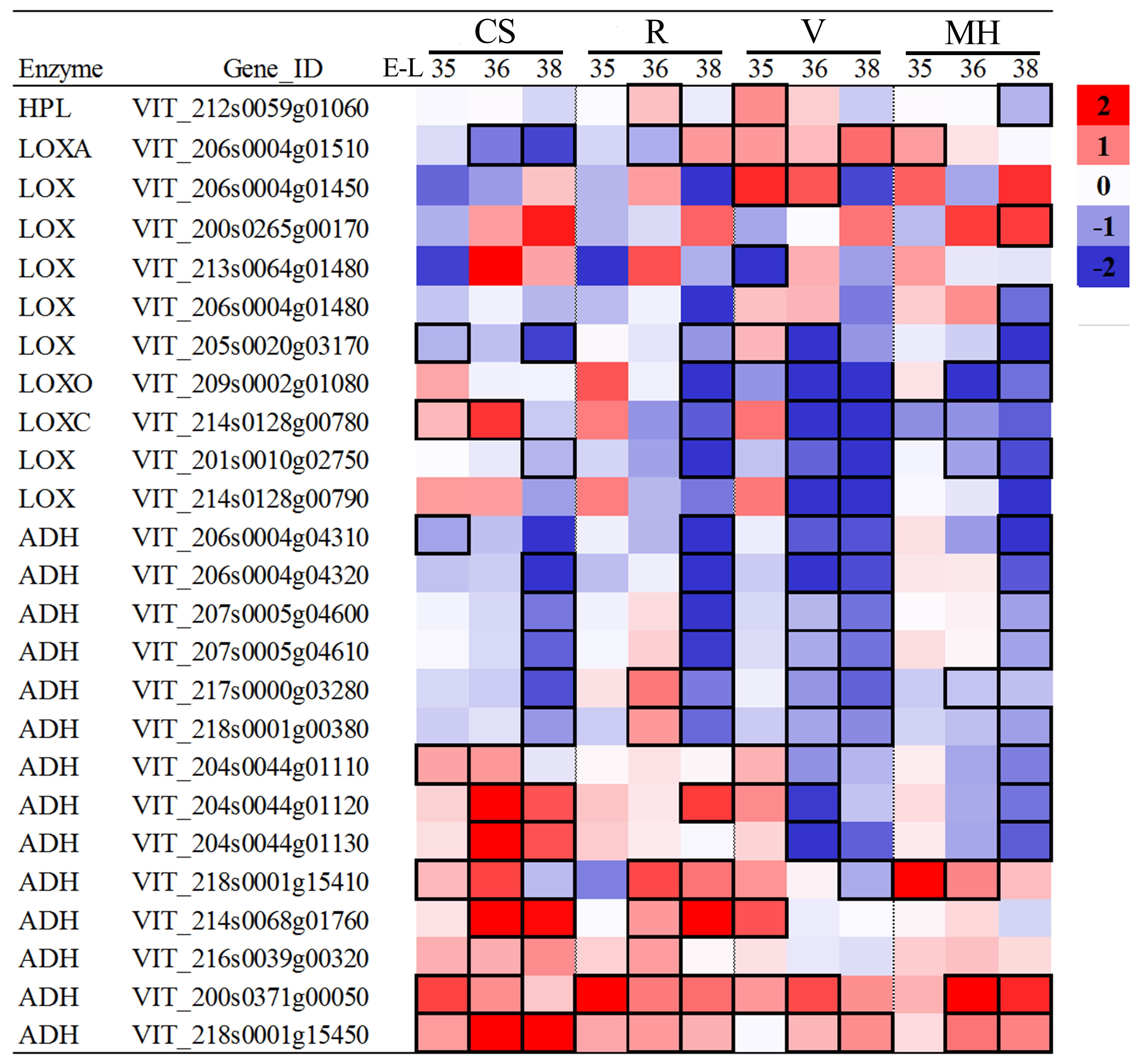


**Supplementary Figure 4.** Effect of the growing season on the gene expression profiles of oxylipin pathway pathways during CS, R, V, and MH grapes development in 2014. Heatmaps showed the log_2_ fold change between seasons (winter season/summer season). Red block indicates higher gene expression in winter season berries. Blue block indicates lower gene expression in winter season berries. Boxes with bold margins indicate differentially expressed genes between summer and winter berries.
